# Abnormal Activation of Tryptophan-Kynurenine Pathway in Women With Polycystic Ovary Syndrome

**DOI:** 10.3389/fendo.2022.877807

**Published:** 2022-06-01

**Authors:** Siyu Wang, Liangshan Mu, Chunmei Zhang, Xiaoyu Long, Yurong Zhang, Rong Li, Yue Zhao, Jie Qiao

**Affiliations:** ^1^ Center for Reproductive Medicine, Department of Obstetrics and Gynecology, Peking University Third Hospital, Beijing, China; ^2^ Key Laboratory of Assisted Reproduction, Ministry of Education, Beijing, China; ^3^ Beijing Key Laboratory of Reproductive Endocrinology and Assisted Reproductive Technology, Beijing, China; ^4^ National Clinical Research Center for Obstetrics and Gynecology (Peking University Third Hospital), Beijing, China; ^5^ Research Units of Comprehensive Diagnosis and Treatment of Oocyte Maturation Arrest, Chinese Academy of Medical Sciences, Beijing, China

**Keywords:** polycystic ovary syndrome, tryptophan metabolism, kynurenine, kynurenic acid, obesity

## Abstract

**Background:**

Women with polycystic ovary syndrome (PCOS) suffer from dysfunctional metabolism and studies have reported increased levels of tryptophan in patients with PCOS. However, the changes of downstream metabolites in tryptophan catabolism pathways remain unclear.

**Methods:**

This is a cross-sectional study that included 200 PCOS patients and 200 control women who were recruited from the Reproductive Medicine Center of Peking University Third Hospital from October 2017 to June 2019. The PCOS patients and the control group were further divided into subtypes of normal weight and overweight/obesity. Fasting blood samples from all subjects were collected on days 2~3 of a natural menstrual cycle or when amenorrhea for over 40 days with follicle diameter not exceeding 10 mm. The plasma levels of tryptophan metabolites were quantitatively determined by the liquid chromatograph mass spectrometer, including tryptophan, serotonin, kynurenine, kynurenic acid, 3-hydroxykynurenine, and quinolinic acid.

**Results:**

The tryptophan-kynurenine pathway was dysregulated in women with PCOS, along with significantly elevated levels of tryptophan, serotonin, kynurenine, kynurenic acid, and quinolinic acid. Moreover, levels of tryptophan, kynurenine, and kynurenic acid were positively correlated with luteinizing hormone, anti-Müllerian hormone, fasting insulin, HOMA-IR. tryptophan, and kynurenine and quinolinic acid had an obvious association with C-reactive protein levels. Furthermore, logistic regression showed that tryptophan, serotonin, kynurenine, kynurenic acid and quinolinic acid were all associated significantly with the increased risk of PCOS with the adjustment for potential confounding factors. Additionally, tryptophan, kynurenine, and kynurenic acid had good diagnostic performances for PCOS, and their combination exhibited higher sensitivity and specificity to diagnostic efficiency, with the area under the ROC curve of 0.824 (95% CI 0.777-0.871), which was comparable to the endocrine indicators.

**Conclusion (s):**

The tryptophan-kynurenine pathway was abnormally activated in PCOS patients.

## Introduction

Polycystic ovary syndrome (PCOS) is the most complicated reproductive endocrine disease in women of childbearing age, which is also recognized as the most common cause of anovulatory infertility (1). Worldwide, the prevalence of PCOS is 4–21% (2, 3), and can reach 5.6% in Chinese women (4). Typical clinical presentations of PCOS include hyperandrogenemia, oligo-or anovulation and polycystic ovaries (5, 6). In addition to reproductive disorders, PCOS patients often suffer from dysfunctional metabolism, such as obesity, insulin resistance, dyslipidemia and metabolic syndrome (7). Furthermore, the elevations of inflammatory cytokines, such as interleukins and chemokines, exacerbate metabolic disturbance in PCOS patients (8). These complications will greatly increase the long-term risk of type 2 diabetes and cardiovascular disease, with an earlier onset age (9, 10). Clinical studies have indicated that rectifying metabolic disorders could improve endocrine and reproductive disorders of PCOS patients (11). Therefore, the exploration of metabolic abnormality and the potential mechanism is the key to detect potential prediction markers and control the incidence of PCOS and complications.

Some metabolomic studies have indicated the imbalance of amino acid metabolism in PCOS, especially the significantly increased levels of aromatic amino acids (tryptophan, phenylalanine, and tyrosine) (12, 13). Tryptophan is one of the essential amino acids necessary for protein synthesis and it is metabolized mainly through kynurenine pathway and serotonin pathway. Under physiological conditions, more than 95% of tryptophan is metabolized by kynurenine (14), with the remainder converted to serotonin through the enterochromaffin cells (15). Tryptophan is firstly catalyzed by the rate-limiting enzyme, indoleamine 2,3-dioxygenase (IDO) or tryptophan 2,3-dioxygenase (TDO), to generate kynurenine. TDO is mainly expressed in the liver and drives tryptophan metabolism under physiological conditions (16); while IDO is widely distributed in whole body tissues outside the liver and its expression is up-regulated under the activation of stress, psychological pressure, and inflammation cytokines (17–20). Kynurenine generates 3-hydroxykynurenine *via* kynurenine monooxygenase and 3-hydroxykynurenine is transformed into 3-hydroxyanthranilic acid and then quinolinic acid through kynureninase and oxidase, respectively (21). In another branch of the kynurenine pathway, kynurenine can be converted into kynurenic acid *via* kynurenine aminotransferases (KAT I-IV).

In existing studies, abnormal activation of the tryptophan-kynurenine pathway is involved in the pathophysiological process of many complex diseases, including tumors, schizophrenia, and metabolic diseases, such as obesity, diabetes, and cardiovascular disease (18, 22–28). In obese adults, serum concentration of kynurenine was positively correlated with BMI and the expression of IDO1 enzymes in adipose tissue increased (29). In a cohort study of diabetic patients, it was found that tryptophan, kynurenine, quinolinic acid, and the ratio of kynurenine to tryptophan (indirect reaction of IDO/TDO enzyme activity) had predictive significance for insulin resistance and the risk of disease after a one-year follow-up (30). Moreover, in the plaque of patients with atherosclerosis, the expression of IDO enzyme in macrophages was up-regulated (31). In view of the multiple metabolic disorders in PCOS, the present study aimed to investigate the changes of the tryptophan-kynurenine pathway in PCOS patients, as well as for the detection of potential metabolic biomarkers for PCOS risk prediction.

## Materials and Methods

### Ethical Approval

This study was approved by the Reproductive Medicine Ethics Committee of Peking University Third Hospital and informed consent of all participants was obtained prior to inclusion in the study.

### Study Population

This is a cross-sectional study included 200 PCOS patients and 200 control women who were recruited from the Reproductive Medicine Center of Peking University Third Hospital from October 2017 to June 2019. PCOS was diagnosed according to the 2003 Rotterdam criteria (6) and met at least two of the following three characteristics: clinical and/or biochemical signs of hyperandrogenemia, oligo-ovaulation or anovulation, and polycystic ovarian ultrasound changes, after exclusion of other causes (such as hypothyroidism, Cushing’s syndrome, congenital adrenal hyperplasia, and hyperprolactinemia). The control group was included from women attending the clinic due to tubal infertility or male factors. All of the control women had normal menstrual cycles, normal ovarian morphology, and did not have clinical or biochemical hyperandrogenemia. All subjects did not take medications known to affect metabolic function or reproductive function within 3 months before enrollment.

### Definition of Metabolic Subtypes

Obesity was defined as body mass index (BMI) ≥ 24kg/m^2^ (32). The index of homeostasis model assessment of insulin resistance (HOMA-IR) was calculated according to the formula: HOMA-IR=fasting insulin (µU/ml) × fasting glucose (mmol/L)/22.5, and insulin resistance was defined as HOMA-IR ≥ 2.69 (33). The diagnostic standard for metabolic syndrome (MetS) is based on the National Cholesterol Education Program Adult Treatment Panel III (NCEP ATP III), which requires at least three of the following features (34): (1) waist circumference (WC) ≥80cm, (2) fasting blood glucose (FPG) ≥ 5.6 mmol/L, (3) systolic blood pressure (SBP) ≥130 mmHg and/or diastolic blood pressure (DBP) ≥85 mmHg, (4) fasting triglycerides (TG) ≥ 1.70 mmol/L, (5) fasting high-density lipoprotein cholesterol (HDL-C) <1.30 mmol/L.

### Sample Collection and Biochemical Measurement

The peripheral blood samples on days 2–3 of a natural menstrual cycle or when amenorrhea for over 40 days with follicle diameter not exceeding 10 mm were collected in the morning after 8 hours of overnight fasting. The serum levels of FPG and fasting serum insulin (FINS) were determined by chemiluminescence method using Immulite 1000 system (DPC, USA). Total cholesterol (T-CHO), TG, low-density lipoprotein cholesterol (LDL-C) and HDL-C were measured by a dry slide enzymatic colorimetric assay. Measurements of serum follicle stimulating hormone (FSH), luteinizing hormone (LH), estradiol, total testosterone (T), androstenedione (AND), and progesterone were performed using a Siemens Immulite 2000 immunoassay system (Siemens Healthcare Diagnostics, USA). Ultrasensitive two-site enzyme-linked immunosorbent assay (Ansh Labs, USA) was used to measure anti-Müllerian hormone (AMH). The plasma samples were centrifuged for the quantitative analyses of metabolites in tryptophan-kynurenine pathway.

### Measurement of Metabolites in Tryptophan-Kynurenine Pathway

Plasma concentrations of tryptophan, serotonin, kynurenine, kynurenic acid, 3-hydroxykynurenine, and quinolinic acid were determined by the liquid Chromatograph (Ultimate3000, Dionex, USA) Mass Spectrometer (API 3200 Q TRAP, AB, USA) (LC-MS) and 50ul of plasma sample was mixed with 150ul of pre-cooled acetonitrile and vortexed for 4 minutes at room temperature. After standing at -20°C for 10 minutes to completely precipitate the protein, the sample was centrifuged at 15000 g for 4 minutes at 4°C and 100ul of the supernatant was taken into the sample bottle of the autosampler to be tested. Tryptophan, serotonin, kynurenine, 3-hydroxykynurenine, and kynurenic acid were tested by +electrospray ionization (ESI) electrospray ion source in multiple reaction monitoring (MRM) scanning mode. The MRM ion pairs of the above metabolites were 205.1/188.1, 177.1/160.0, 209.1/146.1, 225.1/110.1 and 190.0/144.0. The spray voltage was +5500V, the collision gas CAD was Medium, and the atomization temperature was 500°C. The atomization gas was 55PSI, the auxiliary gas was 60PSI, the curtain gas was 20PSI, and the injection voltage was 10V. Chromatographic separation adopted Sapphire C18 chromatographic column (100*4.6mm 5um 100A), and its temperature was 50°C, flow rate was 1ml/min, mobile water phase was distilled water containing 0.1% formic acid, and organic phase was chromatographically pure acetonitrile containing 0.1%. Quinolinic acid adopted -ESI electrospray ion source in MRM multi-reaction monitoring scanning mode. The MRM ion pair of quinolinic acid was 166.1/122.0, and the MRM ion pair of internal standard chloramphenicol was 321.15/152.15. The spray voltage was -4200V, the collision gas CAD was Medium, and the atomization temperature was 500°C. The atomization gas was 55PSI, the auxiliary gas was 60PSI, the curtain gas was 20PSI, and the injection voltage was -10V. The chromatographic separation used a Sapphire C18 column (100*4.6mm 5um 100A), and its temperature was 50°C, the flow rate was 1ml/min, the mobile water phase was distilled water containing 5mM amine acetate, and the organic phase was pure acetonitrile. The chromatographic separation adopted a gradient elution method. Analyst Software version 1.61 was used for mass spectrometry data processing.

### Statistical Analysis

The data were analyzed in SPSS Statistics (version 23.0; IBM, USA). The Kolmogorov-Smirnov test was used to determine whether the continuous variable is normally distributed. Comparisons between PCOS and control groups were performed using an independent sample *t* test and the Mann-Whitney *U* test for normally and non-normally distributed variables, respectively. The data were represented by the median (interquartile range). Spearman’s rank correlation was used to evaluate the correlation between metabolites and baseline endocrine and metabolic indicators. Binary logistic regression was performed to evaluate the correlation between metabolites and the presence of PCOS, before and after adjustment for baseline variables including age, BMI, LH, AND, and AMH. The concentrations of metabolites were further scaled to standard deviation (SD) units for easy comparison of metabolites with large differences in their concentration distributions. Receiver operating characteristic (ROC) curves were prepared for comparison of the diagnostic performance of metabolites in tryptophan-kynurenine pathway and clinical parameters, individually or in combination. All reported confidence interval (CI) values were calculated at the 95% level. *P* < 0.05 was considered statistically significant.

## Results

### Baseline Characteristics of Subjects

The baseline information, endocrine and metabolic indicators between PCOS, and control groups are shown in **Table 1**. Compared with the control group, PCOS patients had significantly increased levels of LH, total testosterone, androstenedione, AMH, fasting insulin, TG, T-CHO and LDL-C, which were in accordance with the typical abnormal characteristics of endocrine and metabolic disorders in PCOS. Also, the levels of uric acid, high sensitivity C-reactive protein (hsCRP) and HOMA-IR, were elevated in PCOS group, indicating the hyperuricemia and inflammatory state in PCOS patients.

**Table 1:** The clinical information of polycystic ovary syndrome (PCOS) and control subjects.

	Control	PCOS	*P* value
Number	200	200	
Age (year)	30.00 (28.00-33.00)	30.00 (28.00-32.00)	0.228
BMI (kg/m2)	23.40 (21.06-25.40)	23.95 (20.84-28.28)	0.064
SBP (mmHg)	120.00 (112.00-127.75)	122.00 (113.50-132.00)	0.101
DBP (mmHg)	76.50 (70.00-81.00)	78.00 (70.50-84.00)	0.173
Prolactin (ng/mL)	10.80 (7.99-14.30)	11.00 (7.76-14.80)	0.876
FSH (mIU/ml)	5.97 (4.71-7.30)	5.69 (4.64-6.74)	0.111
LH (mIU/ml)	3.29 (2.22-4.83)	6.38 (3.73-9.89)	<0.001
LH/FSH	0.55 (0.40-0.79)	1.05 (0.69-1.99)	<0.001
Estradiol (pmol/L)	161.00 (124.50-202.00)	170.00 (141.00-217.00)	0.086
T (nmol/l)	0.69 (0.69-0.70)	0.78 (0.69-1.40)	<0.001
AND (nmol/l)	4.94 (3.47-7.21)	8.77 (5.88-12.60)	<0.001
Progesterone (nmol/L)	0.98 (0.67-1.40)	0.94 (0.68-1.20)	0.577
AMH (ng/ml)	2.88 (1.78-4.28)	7.40 (4.80-11.65)	<0.001
AFC	11.00 (9.00-14.00)	24.00 (18.00-24.00)	<0.001
FPG (mmol/L)	5.10 (4.80-5.30)	5.00 (4.70-5.40)	0.415
FSI (mU/L)	6.72 (4.77-9.84)	11.20 (7.06-17.26)	<0.001
HOMA-IR	1.57 (1.02-2.42)	2.44 (1.51-4.04)	<0.001
T-CHO (mmol/L)	4.26 (3.79-4.81)	4.52 (4.01-5.21)	<0.001
TG (mmol/L)	1.00 (0.73-1.48)	1.24 (0.87-1.83)	0.001
HDL-C (mmol/L)	1.27 (1.11-1.48)	1.26 (1.09-1.48)	0.913
LDL-C (mmol/L)	2.69 (2.29-3.14)	2.95 (2.42-3.59)	0.001
Uric acid (mmol/L)	274.00 (242.00-316.00)	302.00 (254.00-357.00)	<0.001
hsCRP (ng/ml)	0.16 (0.13-0.27)	0.64 (0.24-1.94)	<0.001

BMI, body mass index; SBP, systolic blood pressure; DBP, diastolic blood pressure; FSH, follicle stimulating hormone; LH, luteinizing hormone; T, total testosterone; AND, androstenedione; AMH, anti-Müllerian hormone; AFC, antral follicle counting; FPG, fasting plasma glucose; FSI, fasting serum insulin; HOMA-IR, homeostasis model assessment of insulin resistance; T-CHO, total cholesterol; TG, triglycerides; LDL-C, low-density lipoprotein cholesterol; HDL-C, high-density lipoprotein cholesterol; hsCRP, high sensitivity C-reactive protein. The data were represented by the median (interquartile range). Independent sample t test and the Mann-Whitney U test were used for normally and non-normally distributed variables, respectively.

### Abnormal Activation of Tryptophan-Kynurenine Pathway in PCOS

**Figure 1:**
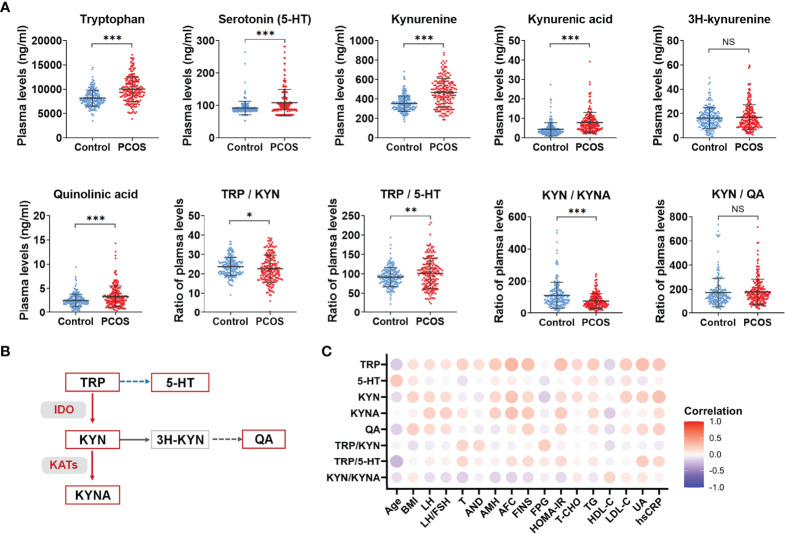
Abnormal activation of tryptophan-kynurenine pathway in PCOS. **(A)** The plasma level changes of metabolites in tryptophan-kynurenine pathway in PCOS patients compared with control groups, ***p < 0.001; **p < 0.01; *p < 0.05; NS, not significant. **(B)** The sketch Map of changes of metabolites and key enzymes in tryptophan-kynurenine pathway in PCOS patients. The red color indicates increased metabolism and activated enzymes, the blue color indicates decreased metabolism, and the grey color indicates that the change trend of this pathway was not yet clear. **(C)** Correlation analysis of differential metabolites levels with the endocrine and metabolic parameters in all subjects. TRP, tryptophan; 5-HT, serotonin; KYN, kynurenine; KYNA, kynurenic acid; QA, quinolinic acid.

The alterations of the metabolite levels and the ratio of the upstream and downstream metabolites in tryptophan-kynurenine pathway are shown in **Figure 1A**. The plasma levels of tryptophan and its metabolites, including serotonin, kynurenine, kynurenic acid, and quinolinic acid, were all elevated in the PCOS group which suggests abnormal activation of the tryptophan catabolism pathway. Also, the ratio of tryptophan to kynurenine (TRP/KYN) was decreased, while the ratio of tryptophan to serotonin (TRP/5-HT) was increased in PCOS group, indicating that the downstream metabolism of tryptophan was more inclined to the direction of the kynurenine pathway. Since the ratio of the up and downstream metabolites could indirectly indicate the enzyme activity involved in the conversion, the increased ratio of kynurenine to tryptophan demonstrated the enhanced activity of IDO/TDO enzyme in PCOS patients (**Figure 1B**). Similarly, a significant decrease in the ratio of kynurenine to kynurenic acid (KYN/KYNA) indirectly indicated an activation of KATs enzyme in the PCOS group (**Figure 1B**).

Previous studies have shown that obesity interacted with the pathophysiological mechanism of PCOS and kynurenine concentration was positively correlated with BMI (35, 36). Although there was no statistically significant difference in BMI between the PCOS and control groups, we further compared the subgroups of normal-weight or overweight/obese population in order to determine whether obesity had an effect on the levels of tryptophan metabolites. The levels of tryptophan, serotonin, kynurenine, kynurenic acid, and quinolinic acid were still significantly increased in PCOS group compared with controls in both normal-weight and overweight/obese population, respectively (**Supplementary Table 1**), indicating that excluding the influence of obesity factors, there was still an interaction between the pathophysiological mechanism of PCOS and abnormal tryptophan metabolism.

Additionally, the abnormal activation of the tryptophan-kynurenine pathway was closely associated with the endocrine and metabolic indicators of PCOS. As **Figure 1C** shows, the levels of tryptophan, kynurenine, and kynurenic acid are positively correlated with LH, AMH, fast insulin levels and HOMA-IR in all subjects and the concentrations of kynurenine and quinolinic acid were positively correlated to BMI. Tryptophan, kynurenic acid, and quinolinic acid had a positive association with TG and negative relation to HDL-C. Interestingly, tryptophan, kynurenine, and quinolinic acid were obviously associated with uric acid and CRP levels. Hence, abnormal activation of the tryptophan-kynurenine pathway might have an impact on the neuroendocrine feedback, insulin sensitivity, and inflammatory state in PCOS patients.

### Identification of Metabolites in Tryptophan-Kynurenine Pathway Associated With PCOS

**Figure 2:**
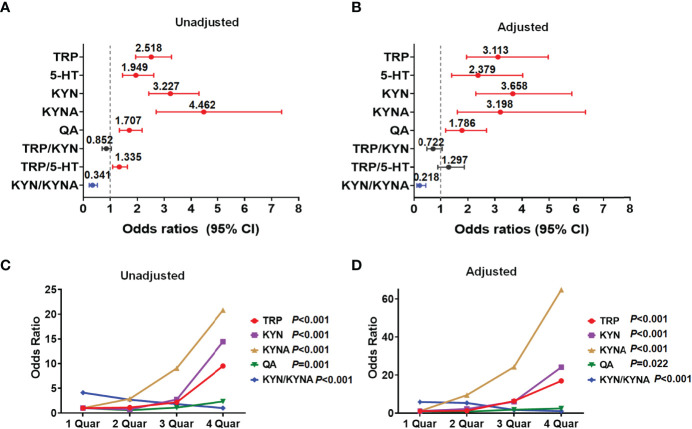
Identification of metabolites in tryptophan-kynurenine pathway associated with PCOS. **(A)** Unadjusted odds ratios (95% CIs) of PCOS per 1 SD change in plasma abundance of each metabolite. **(B)** The odds ratios (95% CIs) of PCOS per 1 SD increase in plasma abundance of each metabolite adjusted for baseline age, BMI, LH, androstenedione, and AMH. **(C, D)** Concentration-effect relationship of metabolites in tryptophan-kynurenine pathway associated with PCOS. The prevalence of PCOS was dramatically raised with the quartiles of TRP, tryptophan; KYN, kynurenine; KYNA,kynurenic acid and QA, quinolinic acid while decreased with quartiles of KYN/KYNA, before **(C)** and after **(D)** adjusting for baseline age, BMI, LH, androstenedione and AMH.

Moreover, we evaluated the potential influence of metabolites in the tryptophan-kynurenine pathway on the occurrence of PCOS and found per SD elevation of the plasma levels of tryptophan metabolites (tryptophan, serotonin, kynurenine, kynurenic acid, and quinolinic acid) and per SD decrease of KYN/KYNA, all were significantly associated with the increased risk of PCOS (**Figure 2A**; **Supplementary Table 2**). With adjustment for baseline age, BMI, and endocrine confounding factors, the correlations between these metabolites and PCOS were still statistically significant and the odds ratios (95% CI) were 3.113 (1.953-4.963), 2.379 (1.406-4.026), 3.658 (2.294-5.833), 3.198 (1.615-6.333) and 1.786 (1.185-2.690), respectively (**Figure 2B**; **Supplementary Table 2**).

Besides, the prevalence of PCOS was dramatically raised with the quartiles of tryptophan, kynurenine, kynurenic acid, and quinolinic acid levels, while notably reduced with the quartiles of KYN/KYNA before and after adjustment for age, BMI, LH, androstenedione, and AMH (**Figures 2C, D**; **Supplementary Table 3**), demonstrating that the abnormal activation of the tryptophan-kynurenine pathway and obviously altered metabolites levels, indeed, profoundly affect the occurrence and development of PCOS.

### Plasma Metabolite Levels of Tryptophan-Kynurenine Pathway Showed Comparable Diagnostic Effect to Endocrine Indicators for PCOS

**Figure 3:**
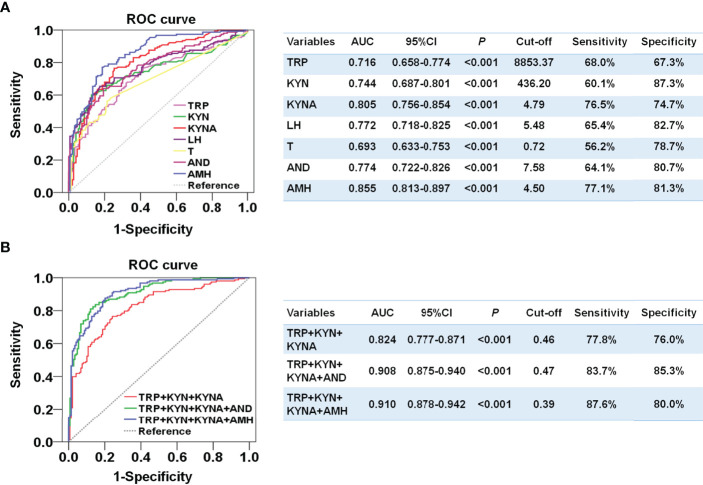
Developing a diagnostic signature of PCOS based on the metabolites in tryptophan-kynurenine pathway. **(A)** Diagnostic potential of TRP, tryptophan; KYN, kynurenine; KYNA,kynurenic acid and the endocrine indicators by ROC analysis to distinguish PCOS from control in all participants. **(B)** Diagnostic potential of the combination of TRP, tryptophan; KYN, kynurenine; KYNA,kynurenic acid with or without the endocrine indicators by ROC analysis to distinguish PCOS from control in all participants. T, total testosterone; AND, androstenedione.

Since the plasma levels of tryptophan, kynurenine, and kynurenic acid were notably enhanced and most correlated with the odds of PCOS, we further compared the ability of these metabolites to distinguish PCOS. Kynurenine exhibited an area under the ROC curve (AUC) of 0.744 (95%CI, 0.658-0.774) and a highest specificity of 87.3%. Kynurenic acid had an AUC of 0.805 (95%CI, 0.756-0.854) with a sensitivity of 76.5% and a specificity of 74.7%, which was better than the diagnostic abilities of LH and androgen and close to that of AMH (**Figure 3A**). Moreover, the combination of tryptophan, kynurenine, and kynurenic acid performed relatively well and distinguished women with PCOS from the controls, and the AUC was 0.824 (95%CI, 0.777-0.871), with well-balanced sensitivity of 77.8% and specificity of 76.0% (**Figure 3B**). Furthermore, the combination of three metabolites and AMH could achieve an AUC of 0.910 (95% CI, 0.875-0.940) with the highest sensitivity of 87.6% and specificity of 80%. These results suggest that the diagnostic performances of metabolites in the tryptophan-kynurenine pathway for PCOS were comparable to the clinical endocrine indicators and could be used as the potential predictive markers of PCOS.

### Determination of Metabolites in Tryptophan-Kynurenine Pathway Associated With the Different Metabolic Disorders in Women With PCOS

**Figure 4:**
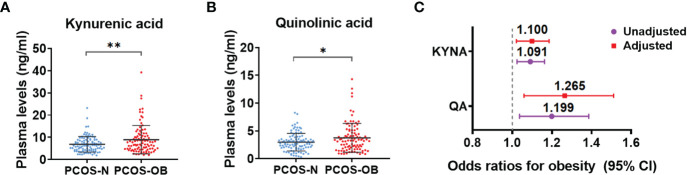
Determination of metabolites in tryptophan-kynurenine pathway associated with the obesity risk in women with PCOS. **(A, B)** The plasma level changes of kynurenic acid **(A)** and quinolinic acid **(B)** in overweight/obese PCOS patients compared with normal weight PCOS women. **p < 0.01; *p < 0.05. **(C)** The odds ratios (95% CIs) of obesity in women with PCOS per 1 SD increase in plasma abundance of KYNA, kynurenic acid and QA, quinolinic acid before and after adjusting for baseline age, LH, androstenedione and AMH.

To determine whether the tryptophan catabolites were associated with the risk of metabolic disorders in PCOS, we further analyzed the alterations of metabolites in the tryptophan-kynurenine pathway in the different subgroups of PCOS patients. The concentrations of kynurenic acid and quinolinic acid were clearly increased in the plasma of overweight/obese PCOS women compared with the subgroup of normal weight PCOS women (**Figures 4A, B**), whereas other metabolites in the tryptophan-kynurenine pathway had no marked alteration (**Supplementary Table 1**). In addition, kynurenic acid and quinolinic acid were found to be associated with the increased odds of obesity in PCOS before and after adjusted for baseline age, LH, androstenedione, and AMH (**Figure 4C**; **Supplementary Table S4**). Conversely, there were no significant alterations in metabolites of the tryptophan-kynurenine pathway between normal weight and obese control subjects (**Supplementary Table 1**), which suggests the alterations of kynurenic acid and quinolinic acid could be specifically used as the predictors of obesity risk in PCOS women but not in non-PCOS women. Unexpectedly, there were no significant changes of metabolites in the tryptophan-kynurenine pathway in the subgroup of PCOS with insulin resistance in comparison to patients with normal insulin sensitivity (**Supplementary Table 5**), as well as in the subgroups of PCOS with and without metabolic syndrome (**Supplementary Table 6**). Thus, the activated tryptophan-kynurenine pathway and dramatically changed metabolites were most affected by the metabolic risk of obesity in PCOS.

## Discussion

Tryptophan is one of the essential mammalian amino acids and the kynurenine pathway is the major metabolic route of tryptophan degradation, which is known to play an important role in the nervous, endocrine, and immune systems (20, 23, 37). In the present study, we detected the plasma levels of tryptophan and its catabolites (serotonin, kynurenine, kynurenic acid, and quinolinic acid) in PCOS patients were significantly increased compared with the control group, as well as the enhanced activities of IDO/TDO and KATs enzymes. Remarkably, elevated metabolites were closely related to increased risk of PCOS and a combination of tryptophan, kynurenine, and kynurenic acid could be used as potential marker to predict the risk of PCOS. In addition, the plasma alterations of kynurenic acid and quinolinic acid most affected the obesity risk in PCOS.

Previous studies have reported the significant change of tryptophan metabolism in several metabolic diseases. The activation of tryptophan metabolism was found in obese adults, meanwhile the ratio of kynurenine to tryptophan was positively correlated with BMI, accompanied by elevated inflammatory cytokines in the plasma (38). Moreover, the elevated level of kynurenine led to the abnormal glucose and lipid metabolism *via* aryl hydrocarbon receptor (AhR) (39, 40). Kynurenic acid, another endogenous ligand of AhR, promoted the occurrence of atherosclerosis by activating the receptor, and thereby could be used as a risk biomarker for the prognosis of atherosclerosis and plaque stability (41). Furthermore, quinolinic acid was involved in the underlying mechanism of diabetes and dyslipidemia by destroying the function of pancreatic cells and adipocytes (29, 42). At present, it is recognized that PCOS is a chronic metabolic disease. Our findings demonstrate the positive association between levels of metabolites in the tryptophan-kynurenine pathway and some metabolic indicators, such as BMI, TG, fast insulin level, and HOMA-IR (**Figure 1C**), indicating the abnormal activation of the kynurenine pathway disturbed the metabolic profile in PCOS. Our study also detected that kynurenic acid and quinolinic acid could aggravate the obesity risk of PCOS patients (**Figure 4**).

Conversely, our results show a positive correlation between the levels of tryptophan, kynurenine, and kynurenic acid and PCOS-related endocrine indexes, LH and AMH, which implies these metabolites had a crucial impact on hypothalamic-pituitary-gonadal (HPG) axis. In previous research on neurological diseases, the initial rate-limiting enzyme IDO was activated, under stress and anxiety, to promote the abnormal metabolism of tryptophan (43). It has been found that tryptophan, kynurenine, and 3-hydroxykynurenine could cross the blood-brain barrier (BBB) and kynurenic acid and quinolinic acid were produced, respectively, in astrocytes and microglia (44, 45). Kynurenic acid had function on neuroprotection and anti-inflammation, and maintained synaptic plasticity (46); quinolinic acid exerted neurotoxicity by inhibiting the reuptake of glutamate, promoting the production of reactive oxygen species, and destroying the BBB (47). Although the mechanism by which its homeostasis was broken is not yet clear, abnormally enhanced levels of kynurenine and its metabolites could induce neuroinflammation (48). In our analysis, the increased plasma concentrations of tryptophan and kynurenine were all notably correlated to the CRP level. Since plasma concentrations of tryptophan and kynurenine could indirectly reflect their contents in brain tissue, the elevation of these two metabolites might lead to the inflammatory reaction of central nervous system and influence the neuroendocrine function in women with PCOS.

In the tryptophan-kynurenine pathway, the initial rate-limiting enzyme is IDO or TDO. In most studies, the plasma ratio of kynurenine to tryptophan was measured as the activity of IDO and TDO. Compared to TDO, IDO is widely distributed as the immunobiologically relevant enzyme that catalyzes the conversion of tryptophan to kynurenine. Clinically, IDO activity was increased in obese adults and positively correlated with BMI (49). The inhibition of IDO activity could improve insulin sensitivity, maintain the intestinal mucosal barrier, reduce endotoxemia and chronic inflammation, and regulate lipid metabolism in both liver and adipose tissues (50). In the current study, the elevation of ratio of kynurenine to tryptophan (**Figure 1A**) intimated IDO and/or TDO might be activated in PCOS patients, which needs further exploration. In addition, because there was no obvious change of ratio of quinolinic acid to kynurenine in PCOS group compared with controls, the increased ratio of kynurenic acid to kynurenine suggests kynurenine metabolism was promoted and shunted to the kynurenic acid pathway in PCOS patients.

Our study also has several limitations. First, due to lack of 40 FINS values, there were 160 patients included in the risk analysis of IR in PCOS (**Supplementary Table 5**). Meanwhile, due to the lack of waist circumference values, it could not be accurately determined if some patients belonged to the MetS group, therefore, 165 were patients included in the risk analysis of MetS in PCOS (**Supplementary Table 6**). Second, it would be ideal to further validate the performance of selected metabolic marker for diagnosing PCOS and predicting the metabolic risks in an independent cohort. Third, it remains unclear whether the abnormal metabolites in the tryptophan-kynurenine pathway contribute directly to the pathogenesis of PCOS or are only a biomarker in the development of disease.

## Conclusions

We investigated systematically the tryptophan catabolism profiles in PCOS and found the abnormal activation of the tryptophan-kynurenine pathway. Our study showed the obvious enhancement of tryptophan, kynurenine, and kynurenic acid in the circulation system; and that they may be considered as biomarkers of PCOS and become potential metabolic intervention targets. Intervention on this pathway, for instance, inhibiting the activities of key enzymes and competitively binding to related receptors, could provide a new strategy for improving metabolic and endocrine disorders in women with PCOS.

## Data Availability Statement

The original contributions presented in the study are included in the article/**Supplementary Material**. Further inquiries can be directed to the corresponding author.

## Ethics Statement

The studies involving human participants were reviewed and approved by Reproductive Medicine Ethics Committee of Peking University Third Hospital. The patients/participants provided their written informed consent to participate in this study.

## Author Contributions

JQ, RL, and YZ conceived and designed this study. SW analyzed the data and wrote the manuscript. LM collected the samples. CZ and XL completed the clinical information. YRZ participated in the sample preparation. All authors took part in the final approval of this study.

## Funding

This study was supported by the National Key Research and Development Project of China (2021YFC2700402), the National Natural Science Foundation of China (82071608, 82001503), the CAMS Innovation Fund for Medical Sciences (2019-I2M-5-001), the China Postdoctoral Science Foundation (2021T140600, 2020M671760), and the Beijing-Tianjin-Hebei Basic Research Cooperation Project (19JCZDJC65000).

## Conflict of Interest

The authors declare that the research was conducted in the absence of any commercial or financial relationships that could be construed as a potential conflict of interest.

## Publisher’s Note

All claims expressed in this article are solely those of the authors and do not necessarily represent those of their affiliated organizations, or those of the publisher, the editors and the reviewers. Any product that may be evaluated in this article, or claim that may be made by its manufacturer, is not guaranteed or endorsed by the publisher.

## References

[1] Escobar-Morreale HF. Polycystic Ovary Syndrome: Definition, Aetiology, Diagnosis and Treatment. Nat Rev Endocrinol (2018) 14(5):270–84. doi: 10.1038/nrendo.2018.24 29569621

[2] Helvaci N Yildiz BO. Polycystic Ovary Syndrome and Aging: Health Implications After Menopause. Maturitas (2020) 139:12–9. doi: 10.1016/j.maturitas.2020.05.013 32747035

[3] Lizneva D Suturina L Walker W Brakta S Gavrilova-Jordan L Azziz R. Criteria, Prevalence, and Phenotypes of Polycystic Ovary Syndrome. Fertil Steril (2016) 106(1):6–15. doi: 10.1016/j.fertnstert.2016.05.003 27233760

[4] Li R Yu G Yang D Li S Lu S Wu X. Prevalence and Predictors of Metabolic Abnormalities in Chinese Women With PCOS: A Cross- Sectional Study. BMC Endocr Disord (2014) 14:76. doi: 10.1186/1472-6823-14-76 25223276 PMC4171713

[5] Nader S. Hyperandrogenism During Puberty in the Development of Polycystic Ovary Syndrome. Fertil Steril (2013) 100(1):39–42. doi: 10.1016/j.fertnstert.2013.03.013 23642453

[6] Azziz R. Controversy in Clinical Endocrinology: Diagnosis of Polycystic Ovarian Syndrome: The Rotterdam Criteria are Premature. J Clin Endocrinol Metab (2006) 91(3):781–5. doi: 10.1210/jc.2005-2153 16418211

[7] Moran LJ Norman RJ Teede HJ. Metabolic Risk in PCOS: Phenotype and Adiposity Impact. Trends Endocrinol Metab (2015) 26(3):136–43. doi: 10.1016/j.tem.2014.12.003 25591984

[8] Shorakae S Ranasinha S Abell S Lambert G Lambert E de Courten B. Inter-Related Effects of Insulin Resistance, Hyperandrogenism, Sympathetic Dysfunction and Chronic Inflammation in PCOS. Clin Endocrinol (Oxf) (2018) 89(5):628–33. doi: 10.1111/cen.13808 29992612

[9] Anagnostis P Tarlatzis BC Kauffman RP. Polycystic Ovarian Syndrome (PCOS): Long-Term Metabolic Consequences. Metabolism (2018) 86:33–43. doi: 10.1016/j.metabol.2017.09.016 29024702

[10] Velez LM Motta AB. Association Between Polycystic Ovary Syndrome and Metabolic Syndrome. Curr Med Chem (2014) 21(35):3999–4012. doi: 10.2174/0929867321666140915141030 25245380

[11] Naderpoor N Shorakae S de Courten B Misso ML Moran LJ Teede HJ. Metformin and Lifestyle Modification in Polycystic Ovary Syndrome: Systematic Review and Meta-Analysis. Hum Reprod Update (2015) 21(5):560–74. doi: 10.1093/humupd/dmv025 26060208

[12] Zhao Y Fu L Li R Wang LN Yang Y Liu NN. Metabolic Profiles Characterizing Different Phenotypes of Polycystic Ovary Syndrome: Plasma Metabolomics Analysis. BMC Med (2012) 10:153. doi: 10.1186/1741-7015-10-153 23198915 PMC3599233

[13] Buszewska-Forajta M Rachon D Stefaniak A Wawrzyniak R Konieczna A Kowalewska A. Identification of the Metabolic Fingerprints in Women With Polycystic Ovary Syndrome Using the Multiplatform Metabolomics Technique. J Steroid Biochem Mol Biol (2019) 186:176–84. doi: 10.1016/j.jsbmb.2018.10.012 30670174

[14] Savitz J. The Kynurenine Pathway: A Finger in Every Pie. Mol Psychiatry (2020) 25(1):131–47. doi: 10.1038/s41380-019-0414-4 PMC6790159 30980044

[15] Bar KJ Kohler S Cruz F Schumann A Zepf FD Wagner G. Functional Consequences of Acute Tryptophan Depletion on Raphe Nuclei Connectivity and Network Organization in Healthy Women. Neuroimage (2020) 207:116362. doi: 10.1016/j.neuroimage.2019.116362 31743788

[16] Sas K Szabo E Vecsei L. Mitochondria, Oxidative Stress and the Kynurenine System, With a Focus on Ageing and Neuroprotection. Molecules (2018) 23(1):191. doi: 10.3390/molecules23010191 PMC6017505 29342113

[17] Oxenkrug GF. Metabolic Syndrome, Age-Associated Neuroendocrine Disorders, and Dysregulation of Tryptophan-Kynurenine Metabolism. Ann N Y Acad Sci (2010) 1199:1–14. doi: 10.1111/j.1749-6632.2009.05356.x 20633104

[18] Chaves Filho AJM Lima CNC Vasconcelos SMM de Lucena DF Maes M Macedo D. IDO Chronic Immune Activation and Tryptophan Metabolic Pathway: A Potential Pathophysiological Link Between Depression and Obesity. Prog Neuropsychopharmacol Biol Psychiatry (2018) 80(Pt C):234–49. doi: 10.1016/j.pnpbp.2017.04.035 28595944

[19] Zang X Zheng X Hou Y Hu M Wang H Bao X. Regulation of Proinflammatory Monocyte Activation by the Kynurenine-AhR Axis Underlies Immunometabolic Control of Depressive Behavior in Mice. FASEB J (2018) 32(4):1944–56. doi: 10.1096/fj.201700853R 29183965

[20] Metghalchi S Ponnuswamy P Simon T Haddad Y Laurans L Clément M. Indoleamine 2,3-Dioxygenase Fine-Tunes Immune Homeostasis in Atherosclerosis and Colitis Through Repression of Interleukin-10 Production. Cell Metab (2015) 22(3):460–71. doi: 10.1016/j.cmet.2015.07.004 26235422

[21] Cervenka I Agudelo LZ Ruas JL. Kynurenines: Tryptophan's Metabolites in Exercise, Inflammation, and Mental Health. Science (2017) 357(6349):eaaf9794. doi: 10.1126/science.aaf9794 28751584

[22] Kindler J Lim CK Weickert CS Boerrigter D Galletly C Liu D. Dysregulation of Kynurenine Metabolism is Related to Proinflammatory Cytokines, Attention, and Prefrontal Cortex Volume in Schizophrenia. Mol Psychiatry (2020) 25(11):2860–72. doi: 10.1038/s41380-019-0401-9 PMC7577855 30940904

[23] Kadriu B Farmer CA Yuan P Park LT Deng ZD Moaddel R. The Kynurenine Pathway and Bipolar Disorder: Intersection of the Monoaminergic and Glutamatergic Systems and Immune Response. Mol Psychiatry (2019) 26(8):4085–95. doi: 10.1038/s41380-019-0589-8 PMC7225078 31732715

[24] Audrito V Manago A Gaudino F Sorci L Messana VG Raffaelli N. NAD-Biosynthetic and Consuming Enzymes as Central Players of Metabolic Regulation of Innate and Adaptive Immune Responses in Cancer. Front Immunol (2019) 10:1720. doi: 10.3389/fimmu.2019.01720 31402913 PMC6671870

[25] Bishnupuri KS Alvarado DM Khouri AN Shabsovich M Chen B Dieckgraefe BK. IDO1 and Kynurenine Pathway Metabolites Activate PI3K-Akt Signaling in the Neoplastic Colon Epithelium to Promote Cancer Cell Proliferation and Inhibit Apoptosis. Cancer Res (2019) 79(6):1138–50. doi: 10.1158/0008-5472.CAN-18-0668 PMC6420842 30679179

[26] Haroon E Welle JR Woolwine BJ Goldsmith DR Baer W Patel T. Associations Among Peripheral and Central Kynurenine Pathway Metabolites and Inflammation in Depression. Neuropsychopharmacology (2020) 45(6):998–1007. doi: 10.1038/s41386-020-0607-1 31940661 PMC7162907

[27] Oxenkrug GF. Increased Plasma Levels of Xanthurenic and Kynurenic Acids in Type 2 Diabetes. Mol Neurobiol (2015) 52(2):805–10. doi: 10.1007/s12035-015-9232-0 PMC4558247 26055228

[28] Agudelo LZ Ferreira DMS Cervenka I Bryzgalova G Dadvar S Jannig PR. Kynurenic Acid and Gpr35 Regulate Adipose Tissue Energy Homeostasis and Inflammation. Cell Metab (2018) 27(2):378–92 e5. doi: 10.1016/j.cmet.2018.01.004 29414686

[29] Favennec M Hennart B Caiazzo R Leloire A Yengo L Verbanck M. The Kynurenine Pathway is Activated in Human Obesity and Shifted Toward Kynurenine Monooxygenase Activation. Obes (Silver Spring) (2015) 23(10):2066–74. doi: 10.1002/oby.21199 26347385

[30] Yu E Papandreou C Ruiz-Canela M Guasch-Ferre M Clish CB Dennis C. Association of Tryptophan Metabolites With Incident Type 2 Diabetes in the PREDIMED Trial: A Case-Cohort Study. Clin Chem (2018) 64(8):1211–20. doi: 10.1373/clinchem.2018.288720 PMC6218929 29884676

[31] Niinisalo P Oksala N Levula M Pelto-Huikko M Jarvinen O Salenius JP. Activation of Indoleamine 2,3-Dioxygenase-Induced Tryptophan Degradation in Advanced Atherosclerotic Plaques: Tampere Vascular Study. Ann Med (2010) 42(1):55–63. doi: 10.3109/07853890903321559 19941414

[32] Consultation WHOE. Appropriate Body-Mass Index for Asian Populations and its Implications for Policy and Intervention Strategies. Lancet (2004) 363(9403):157–63. doi: 10.1016/S0140-6736(03)15268-3 14726171

[33] Xing X-y Yang W-y Yang Z-j. The Diagnostic Significance of Homeostasis Model Assessment of Insulin Resistance in Metabolic Syndrome Among Subjects With Different Glucose Tolerance. Chin J Diabetes (2004) 12(3):182–6.

[34] Expert Panel on Detection E Treatment of High Blood Cholesterol in A. Executive Summary of The Third Report of The National Cholesterol Education Program (NCEP) Expert Panel on Detection, Evaluation, And Treatment of High Blood Cholesterol In Adults (Adult Treatment Panel III). JAMA (2001) 285(19):2486–97. doi: 10.1001/jama.285.19.2486 11368702

[35] Silvestris E de Pergola G Rosania R Loverro G. Obesity as Disruptor of the Female Fertility. Reprod Biol Endocrinol (2018) 16(1):22. doi: 10.1186/s12958-018-0336-z 29523133 PMC5845358

[36] Behboudi-Gandevani S Ramezani Tehrani F Bidhendi Yarandi R Noroozzadeh M Hedayati M Azizi F. The Association Between Polycystic Ovary Syndrome, Obesity, and the Serum Concentration of Adipokines. J Endocrinol Invest (2017) 40(8):859–66. doi: 10.1007/s40618-017-0650-x 28332170

[37] Kim H Chen L Lim G Sung B Wang S McCabe MF. Brain Indoleamine 2,3-Dioxygenase Contributes to the Comorbidity of Pain and Depression. J Clin Invest (2012) 122(8):2940–54. doi: 10.1172/JCI61884 PMC3408737 22751107

[38] Cussotto S Delgado I Anesi A Dexpert S Aubert A Beau C. Tryptophan Metabolic Pathways Are Altered in Obesity and Are Associated With Systemic Inflammation. Front Immunol (2020) 11:557. doi: 10.3389/fimmu.2020.00557 32351500 PMC7174689

[39] Rojas IY Moyer BJ Ringelberg CS Wilkins OM Pooler DB Ness DB. Kynurenine-Induced Aryl Hydrocarbon Receptor Signaling in Mice Causes Body Mass Gain, Liver Steatosis, and Hyperglycemia. Obes (Silver Spring) (2021) 29(2):337–49. doi: 10.1002/oby.23065 PMC10782555 33491319

[40] Moyer BJ Rojas IY Kerley-Hamilton JS Hazlett HF Nemani KV Trask HW. Inhibition of the Aryl Hydrocarbon Receptor Prevents Western Diet-Induced Obesity. Model for AHR Activation by Kynurenine *via* Oxidized-LDL, TLR2/4, TGFbeta, and IDO1. Toxicol Appl Pharmacol (2016) 300:13–24. doi: 10.1016/j.taap.2016.03.011 27020609 PMC4851598

[41] Baumgartner R Berg M Matic L Polyzos KP Forteza MJ Hjorth SA. Evidence That a Deviation in the Kynurenine Pathway Aggravates Atherosclerotic Disease in Humans. J Intern Med (2021) 289(1):53–68. doi: 10.1111/joim.13142 32794238

[42] Hu P Hunt NH Arfuso F Shaw LC Uddin MN Zhu M. Increased Indoleamine 2,3-Dioxygenase and Quinolinic Acid Expression in Microglia and Muller Cells of Diabetic Human and Rodent Retina. Invest Ophthalmol Vis Sci (2017) 58(12):5043–55. doi: 10.1167/iovs.17-21654 PMC5633007 28980000

[43] Heisler JM O'Connor JC. Indoleamine 2,3-Dioxygenase-Dependent Neurotoxic Kynurenine Metabolism Mediates Inflammation-Induced Deficit in Recognition Memory. Brain Behavior Immunity (2015) 50:115–24. doi: 10.1016/j.bbi.2015.06.022 PMC4631688 26130057

[44] Owe-Young R Webster NL Mukhtar M Pomerantz RJ Smythe G Walker D. Kynurenine Pathway Metabolism in Human Blood-Brain-Barrier Cells: Implications for Immune Tolerance and Neurotoxicity. J Neurochem (2008) 105(4):1346–57. doi: 10.1111/j.1471-4159.2008.05241.x 18221377

[45] Platten M Nollen EAA Rohrig UF Fallarino F Opitz CA. Tryptophan Metabolism as a Common Therapeutic Target in Cancer, Neurodegeneration and Beyond. Nat Rev Drug Discovery (2019) 18(5):379–401. doi: 10.1038/s41573-019-0016-5 30760888

[46] Potter MC Elmer GI Bergeron R Albuquerque EX Guidetti P Wu HQ. Reduction of Endogenous Kynurenic Acid Formation Enhances Extracellular Glutamate, Hippocampal Plasticity, and Cognitive Behavior. Neuropsychopharmacology (2010) 35(8):1734–42. doi: 10.1038/npp.2010.39 PMC3055476 20336058

[47] Ferreira FS Schmitz F Marques EP Siebert C Wyse ATS. Intrastriatal Quinolinic Acid Administration Impairs Redox Homeostasis and Induces Inflammatory Changes: Prevention by Kynurenic Acid. Neurotox Res (2020) 38(1):50–8. doi: 10.1007/s12640-020-00192-2 32219734

[48] Heyes MP Saito K Crowley JS Davis LE Demitrack MA Der M. Quinolinic Acid and Kynurenine Pathway Metabolism in Inflammatory and non-Inflammatory Neurological Disease. Brain (1992) 115(5):1249–73. doi: 10.1093/brain/115.5.1249 1422788

[49] Oxenkrug G. Insulin Resistance and Dysregulation of Tryptophan-Kynurenine and Kynurenine-Nicotinamide Adenine Dinucleotide Metabolic Pathways. Mol Neurobiol (2013) 48(2):294–301. doi: 10.1007/s12035-013-8497-4 23813101 PMC3779535

[50] Laurans L Venteclef N Haddad Y Chajadine M Alzaid F Metghalchi S. Genetic Deficiency of Indoleamine 2,3-Dioxygenase Promotes Gut Microbiota-Mediated Metabolic Health. Nat Med (2018) 24(8):1113–20. doi: 10.1038/s41591-018-0060-4 29942089

