# Supplementary material for: Abnormal Activation of Tryptophan-Kynurenine Pathway in Women With Polycystic Ovary Syndrome
Source: Front Endocrinol (Lausanne). 2022 Jun 1;13:877807. doi: 10.3389/fendo.2022.877807 (PMC9199373; doi:10.3389/fendo.2022.877807)
Supplement: Supplementary file 1 [file Table_1.docx]

Supplementary Table 1: The clinical information and plasma levels of metabolites of tryptophan- kynurenine pathway in subjects subtyped by obesity.

|  | Control Normal weight | Control Overweight/obese | PCOS Normal weight | PCOS Overweight/obese | *P* value Con-N vs. PCOS-N | *P* value Con-OB vs. PCOS-OB | *P* value Con-N vs. Con-OB | *P* value PCOS-N vs. PCOS-OB |
| --- | --- | --- | --- | --- | --- | --- | --- | --- |
| Number | 110 | 90 | 101 | 99 |  |  |  |  |
| Age (year) | 29.50 (27.00-32.25) | 31.50 (28.00-34.00) | 30.00 (28.00-32.00) | 29.00 (28.00-33.00) | 0.446 | 0.014 | 0.001 | 0.470 |
| BMI | 21.50 (20.20-22.68) | 25.70 (24.70-27.45) | 20.90 (19.50-22.64) | 28.30 (25.78-31.11) | 0.195 | <0.001 | <0.001 | <0.001 |
| SBP (mmHg) | 119.00 (112.00-126.00) | 121.00 (113.00-130.00) | 117.00 (107.00-126.00) | 128.00 (117.75-138.25) | 0.296 | 0.001 | 0.168 | <0.001 |
| DBP (mmHg) | 75.00 (70.00-80.00) | 77.00 (70.00-81.00) | 75.00 (66.00-80.00) | 81.00 (74.75-86.00) | 0.464 | 0.006 | 0.195 | <0.001 |
| Prolactin (ng/mL) | 11.22 (8.34-13.80) | 10.30 (7.68-14.75) | 12.50 (8.62-16.30) | 9.48 (6.89-13.70) | 0.306 | 0.227 | 0.436 | 0.013 |
| FSH (mIU/ml) | 6.20 (4.95-7.52) | 5.60 (4.46-6.85) | 5.69 (4.57-6.75) | 5.61 (4.89-6.67) | 0.025 | 0.907 | 0.021 | 0.986 |
| LH (mIU/ml) | 3.53 (2.55-5.48) | 2.97 (1.95-4.02) | 6.33 (3.73-9.81) | 6.49 (3.66-9.91) | <0.001 | <0.001 | 0.006 | 0.914 |
| LH/FSH | 0.61 (0.41-0.84) | 0.54 (0.38-0.69) | 1.05 (0.70-1.98) | 1.15 (0.67-2.00) | <0.001 | <0.001 | 0.164 | 0.701 |
| Estradiol (pmol/L) | 170.00 (125.00-201.00) | 155.50 (123.75-206.50) | 168.00 (137.00-209.00) | 173.00 (144.00-226.25) | 0.380 | 0.119 | 0.806 | 0.631 |
| T (nmol/l) | 0.69 (0.69-0.69) | 0.69 (0.69-0.73) | 0.69 (0.69-1.17) | 0.99 (0.69-1.53) | 0.001 | <0.001 | 0.877 | 0.008 |
| AND (nmol/l) | 4.94 (3.48-7.21) | 4.96 (3.46-6.98) | 8.30 (5.39-11.85) | 9.32 (6.53-14.10) | <0.001 | <0.001 | 0.726 | 0.041 |
| Progesterone (nmol/L) | 1.00 (0.69-1.51) | 0.93 (0.64-1.27) | 0.97 (0.71-1.19) | 0.87 (0.64-1.28) | 0.485 | 0.929 | 0.169 | 0.386 |
| AMH (ng/ml) | 3.37 (2.07-4.40) | 2.53 (1.56-4.25) | 7.84 (5.26-12.20) | 6.69 (4.33-10.90) | <0.001 | <0.001 | 0.144 | 0.179 |
| AFC | 11.50 (9.00-14.00) | 11.00 (8.00-14.00) | 24.00 (16.00-24.00) | 24.00 (24.00-24.00) | <0.001 | <0.001 | 0.290 | 0.001 |
| FPG (mmol/L) | 5.00 (4.70-5.30) | 5.20 (4.90-5.50) | 4.90 (4.70-5.15) | 5.20 (4.70-5.70) | 0.204 | 0.845 | 0.006 | 0.002 |
| FINS (mU/L) | 5.82 (4.25-8.72) | 9.82 (5.71-14.70) | 7.13 (5.39-10.43) | 13.73 (10.33-20.69) | 0.084 | 0.002 | 0.009 | <0.001 |
| HOMA-IR | 1.35 (0.98-1.93) | 2.38 (1.30-3.44) | 1.55 (1.07-2.35) | 3.29 (2.35-4.51) | 0.145 | 0.003 | 0.008 | <0.001 |
| T-CHO (mmol/L) | 4.16 (3.79-4.63) | 4.39 (3.85-5.05) | 4.44 (3.93-5.03) | 4.66 (4.22-5.31) | 0.017 | 0.007 | 0.097 | 0.054 |
| TG (mmol/L) | 0.81 (0.62-1.10) | 1.36 (0.98-1.95) | 0.90 (0.76-1.35) | 1.62 (1.12-2.18) | 0.006 | 0.023 | <0.001 | <0.001 |
| HDL-C (mmol/L) | 1.32 (1.21-1.61) | 1.17 (1.02-1.36) | 1.44 (1.21-1.69) | 1.14 (1.02-1.30) | 0.165 | 0.601 | <0.001 | <0.001 |
| LDL-C (mmol/L) | 2.59 (2.18-2.99) | 2.76 (2.38-3.46) | 2.69 (2.17-3.21) | 3.18 (2.67-3.77) | 0.208 | 0.002 | 0.006 | <0.001 |
| UA (mmol/L) | 263.00 (232.00-297.00) | 286.50 (256.00-329.25) | 275.00 (234.50-311.50) | 345.00 (294.50-398.25) | 0.305 | <0.001 | <0.001 | <0.001 |
| hsCRP (ng/ml) | 0.15 (0.10-0.18) | 0.32 (0.15-0.57) | 0.25 (0.16-0.70) | 0.87 (0.33-2.58) | <0.001 | 0.002 | 0.002 | <0.001 |
| TRP (ng/ml) | 7944.55 (6954.03-9384.37) | 7944.55 (7137.84-9093.34) | 9741.77 (8383.64-11661.53) | 9700.93 (8332.58-11886.19) | <0.001 | <0.001 | 0.741 | 0.919 |
| 5-HT (ng/ml) | 88.20 (84.19-96.57) | 87.41 (82.43-93.57) | 94.98 (86.70-123.35) | 96.22 (87.05-123.35) | 0.001 | 0.001 | 0.262 | 0.761 |
| KYN (ng/ml) | 342.51 (291.49-396.12) | 347.71 (303.47-401.85) | 462.23 (369.57-554.88) | 468.47 (353.96-562.17) | <0.001 | <0.001 | 0.506 | 0.985 |
| KYNA (ng/ml) | 3.34 (2.37-5.26) | 3.62 (2.76-4.86) | 6.28 (4.35-8.85) | 7.38 (4.62-11.10) | <0.001 | <0.001 | 0.820 | 0.007 |
| 3H-KYN (ng/ml) | 14.80 (9.62-20.63) | 16.00 (11.58-21.38) | 15.30 (10.95-20.55) | 12.90 (9.09-24.30) | 0.656 | 0.541 | 0.364 | 0.825 |
| QA (ng/ml) | 2.23 (1.77-2.90) | 2.61 (1.90-3.34) | 2.79 (1.96-3.55) | 3.27 (1.83-4.68) | 0.005 | 0.004 | 0.067 | 0.011 |
| TRP/KYN | 23.72 (20.27-27.16) | 23.53 (20.28-26.13) | 21.37 (17.33-27.24) | 22.53 (18.01-28.24) | 0.015 | 0.605 | 0.425 | 0.421 |
| TRP/5-HT | 91.14 (74.62-109.65) | 91.32 (75.86-104.44) | 101.38 (71.21-124.91) | 104.77 (72.14-123.72) | 0.129 | 0.033 | 0.835 | 0.844 |
| KYN/KYNA | 96.09 (58.68-146.61) | 91.06 (70.24-126.46) | 69.06 (51.73-106.63) | 62.34 (39.50-92.83) | 0.005 | <0.001 | 0.883 | 0.032 |
| KYN/QA | 155.26 (107.54-197.76) | 137.37 (105.14-191.50) | 167.53 (125.56-211.74) | 151.12 (98.58-212.14) | 0.181 | 0.757 | 0.337 | 0.081 |

**Abbreviations:** BMI**,** body mass index; SBP, systolic blood pressure; DBP, diastolic blood pressure; FSH, follicle stimulating hormone; LH, luteinizing hormone; T, total testosterone; AND, androstenedione; AMH, anti-Müllerian hormone; AFC, antral follicle counting; FPG, fasting plasma glucose; FSI, fasting serum insulin; HOMA-IR, homeostasis model assessment of insulin resistance; T-CHO, total cholesterol; TG, triglycerides; LDL-C, low-density lipoprotein cholesterol; HDL-C, high-density lipoprotein cholesterol; hsCRP, high sensitivity C-reactive protein; TRP, tryptophan; 5-HT, serotonin; KYN, kynurenine; KYNA, kynurenic acid; 3H-KYN, 3-hydroxykynurenine; QA, quinolinic acid. The data were represented by the median (interquartile range). Independent sample *t* test and the Mann-Whitney *U* test were used for normally and non-normally distributed variables, respectively.
