# Supplementary material for: Abnormal Activation of Tryptophan-Kynurenine Pathway in Women With Polycystic Ovary Syndrome
Source: Front Endocrinol (Lausanne). 2022 Jun 1;13:877807. doi: 10.3389/fendo.2022.877807 (PMC9199373; doi:10.3389/fendo.2022.877807)
Supplement: Supplementary file 2 [file Table_2.docx]

Supplementary Table 2: The odds ratio (OR) of PCOS per SD change of metabolites in tryptophan-kynurenine pathway.

|  | **Unadjusted** | | **Adjusted model 1** | | | **Adjusted model 2** | | | **Adjusted model 3** | | **Adjusted model 4** | | **Adjusted model 5** | | |
| --- | --- | --- | --- | --- | --- | --- | --- | --- | --- | --- | --- | --- | --- | --- | --- |
| Metabolite | OR (95% CI) | *P* | OR (95% CI) | *P* | OR (95% CI) | | *P* | OR (95% CI) | | *P* | OR (95% CI) | *P* | OR (95% CI) | *P* |  |
| TRP | 2.518 (1.941-3.268) | <0.001 | 2.540 (1.947-3.314) | <0.001 | 3.110 (2.205-4.387) | | <0.001 | 2.702 (1.919-3.806) | | <0.001 | 2.444 (1.761-3.393) | <0.001 | 3.113 (1.953-4.963) | <0.001 |  |
| 5-HT | 1.949 (1.455-2.611) | <0.001 | 1.977 (1.456-2.686) | <0.001 | 2.006 (1.393-2.889) | | <0.001 | 2.437 (1.571-3.779) | | <0.001 | 1.935 (1.340-2.793) | <0.001 | 2.379 (1.406-4.026) | 0.001 |  |
| KYN | 3.227 (2.428-4.288) | <0.001 | 3.199 (2.397-4.269) | <0.001 | 3.297 (2.356-4.614) | | <0.001 | 3.597 (2.497-5.180) | | <0.001 | 2.879 (2.028-4.088) | <0.001 | 3.658 (2.294-5.833) | <0.001 |  |
| KYNA | 4.462 (2.702-7.366) | <0.001 | 4.761 (2.807-8.076) | <0.001 | 3.501 (1.999-6.130) | | <0.001 | 4.906 (2.599-9.257) | | <0.001 | 4.146 (2.187-7.857) | <0.001 | 3.198 (1.615-6.333) | 0.001 |  |
| QA | 1.707 (1.337-2.180) | <0.001 | 1.733 (1.339-2.244) | <0.001 | 1.757 (1.281-2.409) | | <0.001 | 1.856 (1.357-2.539) | | <0.001 | 1.916 (1.364-2.693) | <0.001 | 1.786 (1.185-2.690) | 0.006 |  |
| TRP/5-HT | 1.335 (1.088-1.637) | 0.006 | 1.327 (1.076-1.636) | 0.008 | 1.402 (1.076-1.825) | | 0.012 | 1.320 (1.005-1.734) | | 0.046 | 1.297 (0.991-1.697) | 0.058 | 1.297 (0.899-1.873) | 0.164 |  |
| TRP/KYN | 0.852 (0.699-1.039) | 0.115 | 0.863 (0.704-1.058) | 0.157 | 0.859 (0.664-1.111) | | 0.248 | 0.791 (0.605-1.034) | | 0.087 | 0.923 (0.711-1.197) | 0.546 | 0.722 (0.495-1.054) | 0.091 |  |
| KYN/KYNA | 0.341 (0.221-0.524) | <0.001 | 0.338 (0.218-0.523) | <0.001 | 0.344 (0.205-0.576) | | <0.001 | 0.272 (0.153-0.482) | | <0.001 | 0.243 (0.135-0.437) | <0.001 | 0.218 (0.104-0.456) | <0.001 |  |

Model 1: adjust for baseline age and BMI.

Model 2: adjusted for baseline age, BMI and LH.

Model 3: adjusted for baseline age, BMI and androstenedione.

Model 4: adjusted for baseline age, BMI and AMH.

Model 5: adjusted for baseline age, BMI, LH, AND and AMH.

Statistical significance was defined at P-value<0.05.
