# Supplementary material for: Abnormal Activation of Tryptophan-Kynurenine Pathway in Women With Polycystic Ovary Syndrome
Source: Front Endocrinol (Lausanne). 2022 Jun 1;13:877807. doi: 10.3389/fendo.2022.877807 (PMC9199373; doi:10.3389/fendo.2022.877807)
Supplement: Supplementary file 3 [file Table_3.docx]

Supplementary Table 3: Multivariate analysis of metabolites in tryptophan-kynurenine pathway associated with prevalence of PCOS.

| TRP | Concentration category | < 7433.97 | 7433.97-8792.10 | 8792.10-10425.94 | >10425.94 | *P* for trend |
| --- | --- | --- | --- | --- | --- | --- |
|  | Unadjusted | 1.00 (reference) | 1.111 (0.617-1.999) | 2.176 (1.230-3.851) | 9.525 (4.935-18.384) | < 0.001 |
|  | Adjusted model 1 | 1.00 (reference) | 1.027 (0.563-1.871) | 2.109 (1.177-3.779) | 9.492 (4.860-18.539) | < 0.001 |
|  | Adjusted model 2 | 1.00 (reference) | 1.411 (0.659-3.020) | 3.226 (1.559-6.674) | 13.517 (5.997-30.463) | < 0.001 |
|  | Adjusted model 3 | 1.00 (reference) | 1.103 (0.496-2.453) | 3.763 (1.768-8.009) | 11.544 (4.980-26.760) | < 0.001 |
|  | Adjusted model 4 | 1.00 (reference) | 0.848 (0.377-1.907) | 2.307 (1.072-4.964) | 9.334 (3.983-21.874) | < 0.001 |
|  | Adjusted model 5 | 1.00 (reference) | 1.221 (0.392-3.805) | 6.338 (2.155-18.636) | 16.921 (5.170-55.387) | < 0.001 |
| KYN | Concentration category | <310.23 | 310.23-385.19 | 385.19-489.29 | >489.29 | *P* for trend |
|  | Unadjusted | 1.00 (reference) | 0.728 (0.393-1.351) | 2.744 (1.543-4.881) | 14.430 (7.039-29.584) | < 0.001 |
|  | Adjusted model 1 | 1.00 (reference) | 0.698 (0.372-1.308) | 2.732 (1.521-4.906) | 14.194 (6.846-29.429) | < 0.001 |
|  | Adjusted model 2 | 1.00 (reference) | 0.663 (0.310-1.419) | 2.851 (1.424-5.709) | 12.757 (5.651-28.795) | < 0.001 |
|  | Adjusted model 3 | 1.00 (reference) | 1.149 (0.507-2.605) | 3.401 (1.567-7.380) | 18.714 (7.644-45.816) | < 0.001 |
|  | Adjusted model 4 | 1.01 (reference) | 1.049 (0.473-2.323) | 2.916 (1.358-6.259) | 12.577 (5.219-30.305) | < 0.001 |
|  | Adjusted model 5 | 1.00 (reference) | 2.168 (0.726-6.473) | 5.999 (2.058-17.492) | 24.099 (7.439-78.072) | < 0.001 |
| KYNA | Concentration category | <3.12 | 3.12-4.79 | 4.79-7.80 | >7.80 | *P* for trend |
|  | Unadjusted | 1.00 (reference) | 2.867 (1.480-5.554) | 9.067 (4.667-17.616) | 20.814 (10.107-42.864) | < 0.001 |
|  | Adjusted model 1 | 1.00 (reference) | 2.880 (1.476-5.621) | 9.064 (4.636-17.722) | 21.004 (10.071-43.806) | < 0.001 |
|  | Adjusted model 2 | 1.00 (reference) | 2.378 (1.070-5.282) | 8.743 (4.032-18.959) | 20.435 (8.668-48.178) | < 0.001 |
|  | Adjusted model 3 | 1.00 (reference) | 3.419 (1.449-8.063) | 10.693 (4.517-25.315) | 35.079 (13.403-91.812) | < 0.001 |
|  | Adjusted model 4 | 1.00 (reference) | 4.680 (1.794-12.210) | 14.078 (5.427-36.520) | 33.743 (12.252-92.930) | < 0.001 |
|  | Adjusted model 5 | 1.00 (reference) | 9.439 (2.622-33.987) | 24.267 (6.693-87.980) | 64.783 (16.590-252.973) | < 0.001 |
| QA | Concentration category | <1.85 | 1.85-2.56 | 2.56-3.55 | >3.55 | *P* for trend |
|  | Unadjusted | 1.00 (reference) | 0.578 (0.327-1.021) | 1.104 (0.635-1.920) | 2.346 (1.322-4.164) | 0.001 |
|  | Adjusted model 1 | 1.00 (reference) | 0.598 (0.336-1.065) | 1.117 (0.638-1.958) | 2.446 (1.356-4.414) | 0.001 |
|  | Adjusted model 2 | 1.00 (reference) | 0.733 (0.364-1.474) | 1.732 (0.900-3.333) | 2.497 (1.240-5.027) | 0.001 |
|  | Adjusted model 3 | 1.00 (reference) | 0.675 (0.320-1.423) | 1.470 (0.725-2.981) | 2.779 (1.304-5.925) | 0.001 |
|  | Adjusted model 4 | 1.00 (reference) | 0.619 (0.285-1.343) | 1.437 (0.697-2.964) | 2.628 (1.225-5.639) | 0.003 |
|  | Adjusted model 5 | 1.00 (reference) | 0.750 (0.278-2.024) | 1.799 (0.721-4.491) | 2.434 (0.907-6.531) | 0.022 |
| KYN/KYNA | Concentration category | <53.37 | 53.37-79.08 | 79.08-117.78 | >117.78 | *P* for trend |
|  | Unadjusted | 4.125 (2.287-7.439) | 2.705 (1.519-4.814) | 1.810 (1.018-3.219) | 1.00 (reference) | < 0.001 |
|  | Adjusted model 1 | 4.154 (2.276-7.580) | 2.711 (1.509-4.871) | 1.738 (0.971-3.111) | 1.00 (reference) | < 0.001 |
|  | Adjusted model 2 | 3.901 (1.952-7.796) | 2.328 (1.191-4.550) | 1.533 (0.777-3.028) | 1.00 (reference) | < 0.001 |
|  | Adjusted model 3 | 4.744 (2.247-10.020) | 3.664 (1.754-7.657) | 1.707 (0.828-3.520) | 1.00 (reference) | < 0.001 |
|  | Adjusted model 4 | 5.589 (2.459-12.704) | 3.494 (1.564-7.806) | 1.868 (0.844-4.134) | 1.00 (reference) | < 0.001 |
|  | Adjusted model 5 | 5.835 (2.146-15.863) | 5.317 (1.932-14.633) | 1.717 (0.641-4.601) | 1.00 (reference) | < 0.001 |

Model 1: adjust for baseline age and BMI.

Model 2: adjusted for baseline age, BMI and LH.

Model 3: adjusted for baseline age, BMI and androstenedione.

Model 4: adjusted for baseline age, BMI and AMH.

Model 5: adjusted for baseline age, BMI, LH, androstenedione and AMH.

Statistical significance was defined at *P*-value<0.05.
