# Supplementary material for: Abnormal Activation of Tryptophan-Kynurenine Pathway in Women With Polycystic Ovary Syndrome
Source: Front Endocrinol (Lausanne). 2022 Jun 1;13:877807. doi: 10.3389/fendo.2022.877807 (PMC9199373; doi:10.3389/fendo.2022.877807)
Supplement: Supplementary file 4 [file Table_4.docx]

Supplementary Table 4: Analysis of metabolites in tryptophan-kynurenine pathway associated with overweight/obese in PCOS.

|  |  | OR (95% CI) | *P*-value |
| --- | --- | --- | --- |
| KYNA | Unadjusted | 1.091 (1.024-1.163) | 0.007 |
|  | Adjusted model 1 | 1.092 (1.025-1.163) | 0.007 |
|  | Adjusted model 2 | 1.093 (1.022-1.169) | 0.009 |
|  | Adjusted model 3 | 1.094 (1.019-1.174) | 0.013 |
|  | Adjusted model 4 | 1.082 (1.017-1.152) | 0.013 |
|  | Adjusted model 5 | 1.100 (1.021-1.186) | 0.013 |
| QA | Unadjusted | 1.199 (1.037-1.386) | 0.014 |
|  | Adjusted model 1 | 1.201 (1.038-1.390) | 0.014 |
|  | Adjusted model 2 | 1.204 (1.024-1.416) | 0.024 |
|  | Adjusted model 3 | 1.253 (1.059-1.482) | 0.009 |
|  | Adjusted model 4 | 1.183 (1.021-1.371) | 0.025 |
|  | Adjusted model 5 | 1.265 (1.059-1.511) | 0.010 |

Model 1: adjust for baseline age.

Model 2: adjusted for baseline age and LH.

Model 3: adjusted for baseline age and androstenedione.

Model 4 adjusted for baseline age and AMH.

Model 5: adjusted for baseline age, LH, androstenedione and AMH.

Statistical significance was defined at *P*-value<0.05.
