# Supplementary material for: Abnormal Activation of Tryptophan-Kynurenine Pathway in Women With Polycystic Ovary Syndrome
Source: Front Endocrinol (Lausanne). 2022 Jun 1;13:877807. doi: 10.3389/fendo.2022.877807 (PMC9199373; doi:10.3389/fendo.2022.877807)
Supplement: Supplementary file 5 [file Table_5.docx]

Supplementary Table 5: The clinical information and plasma levels of metabolites of tryptophan- kynurenine pathway in PCOS patients with and without insulin resistance (IR).

|  | PCOS without IR | PCOS with IR | *P* value |
| --- | --- | --- | --- |
| Number | 82 | 78 |  |
| Age (year) | 29.50 (28.00-31.00) | 29.00 (27.00-31.00) | 0.237 |
| BMI | 22.02 (19.98-25.25) | 28.00 (24.54-31.20) | < 0.001 |
| SBP (mmHg) | 116.00 (106.00-127.00) | 128.00 (120.50-140.00) | < 0.001 |
| DBP (mmHg) | 73.00 (65.25-80.00) | 82.00 (76.00-87.50) | < 0.001 |
| Prolactin (ng/mL) | 11.00 (8.47-14.78) | 8.80 (6.64-13.10) | 0.025 |
| FSH (mIU/ml) | 5.74 (4.61-6.70) | 5.47 (4.78-6.49) | 0.617 |
| LH (mIU/ml) | 7.58 (5.21-11.80) | 6.85 (4.62-9.43) | 0.175 |
| LH/FSH | 1.33 (0.89-2.36) | 1.16 (0.79-1.91) | 0.215 |
| Estradiol (pmol/L) | 172.00 (146.00-232.00) | 172.00 (139.00-204.50) | 0.635 |
| T (nmol/l) | 0.84 (0.69-1.52) | 0.84 (0.69-1.42) | 0.972 |
| AND (nmol/l) | 9.42 (6.09-13.20) | 9.03 (6.48-13.80) | 0.853 |
| Progesterone (nmol/L) | 0.98 (0.66-1.19) | 0.80 (0.65-1.32) | 0.493 |
| AMH (ng/ml) | 9.50 (5.91-13.96) | 5.99 (4.13-10.39) | < 0.001 |
| AFC | 24.00 (19.25-24.00) | 24.00 (24.00-24.00) | 0.101 |
| FPG (mmol/L) | 4.90 (4.60-5.10) | 5.40 (4.98-5.80) | < 0.001 |
| FINS (mU/L) | 7.23 (5.48-9.20) | 17.26 (13.38-21.92) | < 0.001 |
| HOMA-IR | 1.52 (1.15-2.06) | 4.07 (3.10-5.43) | < 0.001 |
| T-CHO (mmol/L) | 4.68 (4.11-5.51) | 4.63 (4.11-5.34) | 0.683 |
| TG (mmol/L) | 1.05 (0.78-1.46) | 1.79 (1.24-2.35) | < 0.001 |
| HDL-C (mmol/L) | 1.41 (1.16-1.70) | 1.13 (1.01-1.28) | < 0.001 |
| LDL-C (mmol/L) | 2.97 (2.35-3.62) | 3.05 (2.63-3.64) | 0.267 |
| UA (mmol/L) | 281.00 (237.75-330.75) | 351.50 (297.00-411.25) | < 0.001 |
| hsCRP (ng/ml) | 0.34 (0.18-0.87) | 0.86 (0.28-2.82) | 0.003 |
| TRP (ng/ml) | 9363.95 (8506.18-11569.63) | 9803.04 (7791.37-11763.65) | 0.712 |
| 5-HT (ng/ml) | 96.22 (86.74-123.35) | 98.15 (80.00-140.98) | 0.904 |
| KYN (ng/ml) | 473.68 (352.40-541.35) | 467.43 (352.40-575.18) | 0.703 |
| KYNA (ng/ml) | 6.41 (4.52-8.85) | 7.99 (4.89-10.70) | 0.095 |
| 3H-KYN (ng/ml) | 16.90 (10.05-23.63) | 12.85 (9.25-20.30) | 0.132 |
| QA (ng/ml) | 3.02 (1.55-4.37) | 3.17 (1.75-4.69) | 0.455 |
| TRP/KYN | 22.06 (17.93-28.81) | 21.63 (17.84-28.31) | 0.981 |
| TRP/5-HT | 99.32 (74.44-124.08) | 104.83 (62.55-126.25) | 0.997 |
| KYN/KYNA | 67.40 (42.42-107.97) | 58.68 (44.23-83.83) | 0.200 |
| KYN/3H-KYN | 26.95 (16.76-49.63) | 32.59 (22.60-53.88) | 0.169 |
| TRP/QA | 3427.98 (2525.36-5437.75) | 3365.23 (2146.96-5663.33) | 0.695 |
| KYN/QA | 161.67 (114.92-211.56) | 159.13 (104.11-221.78) | 0.598 |
| 3H-KYN/QA | 5.84 (3.11-11.75) | 4.80 (2.46-10.67) | 0.185 |

**Abbreviations:** BMI**,** body mass index; SBP, systolic blood pressure; DBP, diastolic blood pressure; FSH, follicle stimulating hormone; LH, luteinizing hormone; T, total testosterone; AND, androstenedione; AMH, anti-Müllerian hormone; AFC, antral follicle counting; FPG, fasting plasma glucose; FSI, fasting serum insulin; HOMA-IR, homeostasis model assessment of insulin resistance; T-CHO, total cholesterol; TG, triglycerides; LDL-C, low-density lipoprotein cholesterol; HDL-C, high-density lipoprotein cholesterol; hsCRP, high sensitivity C-reactive protein; TRP, tryptophan; 5-HT, serotonin; KYN, kynurenine; KYNA, kynurenic acid; 3H-KYN, 3-hydroxykynurenine; QA, quinolinic acid. The data were represented by the median (interquartile range). Independent sample *t* test and the Mann-Whitney *U* test were used for normally and non-normally distributed variables, respectively.
