# Supplementary material for: Abnormal Activation of Tryptophan-Kynurenine Pathway in Women With Polycystic Ovary Syndrome
Source: Front Endocrinol (Lausanne). 2022 Jun 1;13:877807. doi: 10.3389/fendo.2022.877807 (PMC9199373; doi:10.3389/fendo.2022.877807)
Supplement: Supplementary file 6 [file Table_6.docx]

Supplementary Table 6: The clinical information and plasma levels of metabolites of tryptophan- kynurenine pathway in PCOS patients with and without metabolic syndrome (MetS).

|  | PCOS without MetS | PCOS with MetS | *P* value |
| --- | --- | --- | --- |
| Number | 104 | 61 |  |
| Age (year) | 30.00 (28.00-32.00) | 29.00 (27.00-32.00) | 0.212 |
| BMI | 21.89 (19.60-24.18) | 29.02 (25.66-31.04) | < 0.001 |
| SBP (mmHg) | 117.00 (107.00-125.00) | 131.00 (121.75-144.25) | < 0.001 |
| DBP (mmHg) | 75.00 (68.00-80.00) | 85.00 (77.50-90.00) | < 0.001 |
| Prolactin (ng/mL) | 11.95 (8.85-15.80) | 8.80 (6.64-12.75) | 0.004 |
| FSH (mIU/ml) | 5.89 (4.93-6.88) | 5.43 (4.62-6.54) | 0.096 |
| LH (mIU/ml) | 7.08 (4.33-11.30) | 6.91 (4.43-9.14) | 0.414 |
| LH/FSH | 1.24 (0.80-2.13) | 1.29 (0.80-2.00) | 0.815 |
| Estradiol (pmol/L) | 169.50 (140.25-216.75) | 169.00 (137.50-204.50) | 0.934 |
| T (nmol/l) | 0.72 (0.69-1.45) | 0.97 (0.69-1.42) | 0.181 |
| AND (nmol/l) | 8.33 (5.43-12.00) | 9.10 (6.66-14.10) | 0.150 |
| Progesterone (nmol/L) | 0.94 (0.68-1.19) | 0.93 (0.65-1.47) | 0.944 |
| AMH (ng/ml) | 7.57 (5.36-13.38) | 5.86 (3.93-10.55) | 0.035 |
| AFC | 24.00 (17.50-24.00) | 24.00 (24.00-27.00) | < 0.001 |
| FPG (mmol/L) | 4.90 (4.70-5.10) | 5.50 (5.05-5.85) | < 0.001 |
| FINS (mU/L) | 7.67 (5.82-11.25) | 17.23 (11.97-21.24) | < 0.001 |
| HOMA-IR | 1.62 (1.20-2.38) | 4.07 (2.87-5.42) | < 0.001 |
| T-CHO (mmol/L) | 4.48 (3.98-4.99) | 4.77 (4.28-5.41) | 0.040 |
| TG (mmol/L) | 1.01 (0.77-1.34) | 1.90 (1.48-2.75) | < 0.001 |
| HDL-C (mmol/L) | 1.46 (1.28-1.69) | 1.11 (1.00-1.25) | < 0.001 |
| LDL-C (mmol/L) | 2.74 (2.23-3.24) | 3.28 (2.69-3.91) | < 0.001 |
| UA (mmol/L) | 280.50 (238.00-322.25) | 358.00 (307.50-442.00) | < 0.001 |
| hsCRP (ng/ml) | 0.36 (0.18-0.86) | 1.01 (0.30-2.71) | 0.001 |
| TRP (ng/ml) | 9415.00 (8501.07-11472.62) | 9700.93 (7587.14-11804.49) | 0.919 |
| 5-HT (ng/ml) | 92.43 (86.04-109.17) | 105.73 (70.49-140.98) | 0.252 |
| KYN (ng/ml) | 458.06 (353.96-540.83) | 443.49 (312.32-562.17) | 0.938 |
| KYNA (ng/ml) | 6.77 (4.71-9.75) | 7.80 (4.82-10.14) | 0.395 |
| 3H-KYN (ng/ml) | 14.95 (9.76-22.48) | 12.60 (9.30-18.85) | 0.241 |
| QA (ng/ml) | 2.83 (1.84-4.10) | 2.65 (1.72-4.23) | 0.894 |
| TRP/KYN | 21.56 (17.25-28.57) | 21.87 (17.68-29.84) | 0.715 |
| TRP/5-HT | 98.44 (75.10-125.89) | 99.44 (62.51-116.09) | 0.378 |
| KYN/KYNA | 64.58 (44.90-96.48) | 58.59 (43.76-91.34) | 0.328 |
| KYN/3H-KYN | 30.37 (18.64-47.00) | 32.31 (21.62-54.08) | 0.379 |
| TRP/QA | 3244.94 (2525.76-5377.30) | 3586.09 (2165.81-5826.51) | 0.821 |
| KYN/QA | 160.31 (119.11-211.74) | 170.77 (95.07-233.67) | 0.931 |
| 3H-KYN/QA | 5.58 (2.92-10.85) | 4.39 (2.55-11.20) | 0.412 |

**Abbreviations:** BMI**,** body mass index; SBP, systolic blood pressure; DBP, diastolic blood pressure; FSH, follicle stimulating hormone; LH, luteinizing hormone; T, total testosterone; AND, androstenedione; AMH, anti-Müllerian hormone; AFC, antral follicle counting; FPG, fasting plasma glucose; FSI, fasting serum insulin; HOMA-IR, homeostasis model assessment of insulin resistance; T-CHO, total cholesterol; TG, triglycerides; LDL-C, low-density lipoprotein cholesterol; HDL-C, high-density lipoprotein cholesterol; hsCRP, high sensitivity C-reactive protein; TRP, tryptophan; 5-HT, serotonin; KYN, kynurenine; KYNA, kynurenic acid; 3H-KYN, 3-hydroxykynurenine; QA, quinolinic acid. The data were represented by the median (interquartile range). Independent sample *t* test and the Mann-Whitney *U* test were used for normally and non-normally distributed variables, respectively.
